# Opinion and perspectives on artificial intelligence biology

**DOI:** 10.1002/qub2.70061

**Published:** 2026-10-20

**Authors:** Xuegong Zhang, Xiaowo Wang, Kai Ye, Dongbo Bu, Fangqing Zhao, Ge Gao

**Affiliations:** ^1^ MOE Key Lab of Bioinformatics, Department of Automation and BNRIST Tsinghua University Beijing China; ^2^ Center for Systems and Synthetic Biology School of Life Sciences and School of Medicine Tsinghua University Beijing China; ^3^ School of Automation Science and Engineering, Faculty of Electronic and Information Engineering Xi’an Jiaotong University Xi’an China; ^4^ University of Chinese Academy of Sciences Beijing China; ^5^ Institute for Computer Science and Technology Chinese Academy of Sciences Beijing China; ^6^ State Key Laboratory of Animal Biodiversity Conservation and Integrated Pest Management Institute of Zoology Chinese Academy of Sciences Beijing China; ^7^ State Key Laboratory of Gene Function and Modulation Research Biomedical Pioneering Innovative Center (BIOPIC) & Beijing Advanced Innovation Center for Genomics (ICG) School of Life Sciences Center for Bioinformatics (CBI) Peking University Beijing China

## 1 INTRODUCTION

As the integration of artificial intelligence (AI) with life sciences is accelerating, its conceptual boundaries, methodological foundations, and future trajectories remain actively debated. Drawing on perspectives from frontline researchers active in AI research in multiple sub‐fields of biology, this article examines the evolution, current states, and future directions of AI biology. We identify a paradigm shift from observation‐driven inquiry toward discovery‐ and design‐oriented research. We discuss the relationship between predictive modeling and mechanistic understanding, the relative importance of terminology, and the future relation of experiments and computation. We argue that future AI biology will require not only algorithmic adaptation or data accumulation, but more fundamental innovations in AI models, biological experiments, and construction of closed‐loop systems that couple prediction with feedback. Furthermore, we appeal to the cultivation of next‐generation scientists who are better equipped with critical thinking and can navigate both domains with equal fluency. This article is intended for a broad range of readers who may care about, work on, prepare for, evaluate, or sponsor future AI biology studies.

## 2 THE EVOLUTION OF AI IN BIOLOGICAL RESEARCH

The 21st century has witnessed the convergence of two transformative revolutions: the rise of AI and the digital transformation of biology. In information sciences, the era of big data and advances in deep learning (DL) revolutionized computer vision (CV) and natural language processing (NLP), ultimately leading to the emergence of large language models (LLMs) and generative AI [1, 2]. In parallel, the completion of the Human Genome Project, together with the rapid development of high‐throughput sequencing, single‐cell technologies, and advanced imaging platforms, has transformed biology from a largely observational discipline into a data‐rich and quantitative science [3]. The convergence of these two revolutions has given rise to a new era of AI‐powered biology, in which AI is evolving from a tool for data analysis toward a framework for representing, predicting, and designing biological systems [4].

The term “Artificial Intelligence (AI)” had carried different meanings in history. When it was coined at the 1956 Dartmouth Summer Workshop, the meaning differed substantially from today’s AI [5]. Today’s AI, characterized by LLM‐based chatbots and generative models, is rooted in machine learning (ML) rather than the original symbolic AI. Although “AI for Science” is a recent term, ML—including DL, a more advanced branch—has been applied to biological problems for decades. For example, just 1 year after the backpropagation‐based multi‐layer perceptron was published, it was used to predict protein secondary structures from its amino acid sequences [6]. As ML is now viewed as a core component of AI, we use “AI” broadly throughout this article to encompass both modern DL/LLM‐based models and more traditional ML approaches.

AI applications spanning diverse biological domains share a consistent pattern: whenever new data become available for studying biologically relevant properties, computational biologists quickly build models to analyze the data and predict the properties. Early approaches relied on statistical models, rule‐based algorithms, and handcrafted features, for example, Markov models for sequence analysis, motif‐based approaches for regulatory genomics, co‐evolutionary statistics for structural inference, and pattern recognition methods for transcriptomics and functional genomics. As data grew, ML models such as neural networks (NNs) and support vector machines were adopted for classification and prediction. More recently, end‐to‐end deep representation learning, foundation models, and generative models have become widespread in biology, used not only to predict labels, but also to generate high‐dimensional outputs, for example, gene expression responses to perturbations.

Today, the frontier is expanding from prediction and generation to active design. For example, in regulatory and functional genomics, models have moved from predicting chromatin accessibility and transcription‐factor binding to generating synthetic promoters with targeted expression patterns, or prioritizing gene combos that can drive desired cell‐state transitions [7, 8]. In structural biology, the field has progressed from predicting protein structures of given amino acid sequences and inferring residue contacts to de novo protein design with specified structures and functions [9, 10]. In genome informatics, the emphasis is expanding from annotating reference genomes to interpreting population‐scale variation, pangenomes, and cross‐species evolutionary patterns [11, 12, 13]. Researchers in single‐cell and spatial omics are leveraging AI for tasks beyond clustering and visualization, toward forecasting cell fate variation, inferring causal factors and regulatory networks, predicting cell‐cell interactions, and reconstructing tissue organizations [14, 15, 16, 17, 18, 19].

A major driver underpinning the evolution is the technological advancements of high‐throughput sequencing and imaging, which have generated data of unprecedented scale and complexity. Many biologists had recognized that modern molecular genetics had effectively transformed biology into an information science [20]. The data growth has promoted the transfer and adaptation of large AI models from CV and NLP to biological domains. At the same time, representation of biological data itself has shifted: genomic differences can now be cast into image‐like spaces, three‐dimensional interactions into spatial structures, gene expressions and cell transcriptomics into context‐aware semantic embeddings, and molecular sequences into high‐dimensional vectors [21, 22, 23, 24]. Such representations enable quantitative computation of biological problems that were previously impossible to formulate—a key factor behind the growing momentum of AI for Science.

## 3 THE ON‐GOING REVOLUTION

### 3.1 A paradigm shift in the making

There is a broad agreement that AI is catalyzing a genuine paradigm shift in biology. However, the nature of this shift is nuanced. Our vision is that, it does not imply the replacement of experimental science, but rather redefines the scale, unit, and workflow of biological inquiry. Traditional research often proceeded from single genes, pathways, or phenotypes. The emerging AI models tend to operate at the systems level—whole genomes, entire cell atlases, complete developmental trajectories, and deep evolutionary histories. The interaction of AI with modern synthetic biology further empowers the trend to treat biological systems as engineerable entities.

One characterization of this shift is the transition from an observation‐driven discipline to a model‐guided and design‐oriented discipline. AI does not eliminate the need for experiments, but it fundamentally changes how experiments are conceived. Predictive modeling and simulation can reduce the experimental search space by orders of magnitude, whereas automated experimentation enables rapid validation and interactive refinement. Over time, this may render certain areas of biology more akin to engineering, in which the goal is not only to understand how systems work, but to design, modify, and construct them for applications in medicine, agriculture, and biomanufacturing.

The shift may be viewed from an epistemological lens. Beyond recording observations, biology has historically been approached through multiple intellectual traditions—physicists seeking first principles, statisticians building explicit data models, and, most recently, computational biologists employing AI modeling that learns empirical distributions directly from large‐scale data without strong prior assumptions. The ascendancy of this third approach represents a break from earlier paradigms that replied on human‐crafted rules or assumptions. The new approach will not displace existing approaches, but will rather work in complement with them: It expands what can be asked and enables scientists to investigate questions that were previously out of reach. Whether this constitutes a permanent reconfiguration or a transitional phase in the history of human science remains an open question, but its immediate impact on practice is undeniable.

### 3.2 Conceptual framing: “AI for Biology” versus “AI Biology”

The terminology used to describe this rapidly evolving interdisciplinary space carries more than semantic weight. Many researchers distinguish between “AI for Biology” (or “AI for Life Sciences”) and “AI Biology.” The former is generally understood as a tool‐oriented framing, in which biology provides an object of study and AI provides instruments. The latter suggests a more integrated discipline in which AI is embedded in the research process itself—participating in hypothesis generation, system modeling, and biological engineering design.

Proponents of the “AI Biology” framing argue that it encourages a more ambitious research agenda: not merely applying AI tools to biological datasets, but asking whether living systems can be modeled as complex systems that are learnable, predictable, verifiable, and designable, and whether AI can help discover new biological principles. Others caution that conceptual terminology, while maybe useful for community building and branding, should not be privileged over substantive progress. The two terms are not mutually exclusive, but refer to overlapping territories. Recall the early history of AI: The terminology “AI” proposed at the Dartmouth Workshop defeated the competing terminology “Cybernetics” named in a series of 10 meetings in 1940s and 1950s, but it was the key principles of the latter that had led to the flourishing of today’s AI [5]. In AI Biology at this stage, it may not be necessary or beneficial to put much emphasis on the terminology. Especially, funding agencies and evaluators should not be distracted by the surface value of terminology.

Along with those two terms, there are other related terms. One is “Biology for AI.” Researchers use it to refer to the need and potential of using biological principles and problems to drive inventions in AI itself. There is much research on “brain‐inspired AI,” but biology is not only about the brain. There are many “smart” mechanisms even in simple unicellular organisms. Some fundamental works in AI, such as perceptrons, Hopfield nets, and convolutional neural networks, had been inspired by early neurobiology discoveries. But the recent more advanced understandings of biology have not yet played a role in AI innovations. There should be large research space for “Biology for AI.”

Another term “AI for Scientists” is only coined recently by researchers when referring to agentic AI tools or “AI Scientists.” AI agents are designed to help scientists in their efficiency, to enhance or expand their scientific reasoning, or to work at some level as autonomous scientists. They are distinct from the canonical “AI for Science” in the sense that their object of study is the scientific work itself instead of biological questions.

All these arguments about the terms make great sense as long as they are used to help clarifying research thoughts and strategies, instead of claiming “terminology innovations.”

## 4 LOOKING FORWARD TO FUTURE AI BIOLOGY

### 4.1 Future directions: Biological and computational perspectives

#### 4.1.1 From the viewpoint of biology

A shared aspiration across domains is to move “reading” biological information to “understanding” and ultimately “designing” biological systems. In genomics, this implies predicting gene structures, regulatory logic, and functional consequences directly from sequences, even for non‐model organisms with limited experimental data, and to design sequences for designated functions. It also means moving beyond single reference genomes to embrace population diversity, structural variation, and evolutionary comparison. In gene regulation, the frontier lies in achieving quantitative spatiotemporal control of expression and phenotypes through computational design of regulatory factors, regulatory sequences, or perturbations. In single‐cell and spatial biology, the goal is to integrate multi‐omics data to build AI models of cells, tissues, and organs for interpretation, prediction, and manipulation in silico, depicted as AI virtual cells (AIVCs) and virtual tissues/organs [25, 26].

#### 4.1.2 From the viewpoint of AI

Biological systems present challenges that differ fundamentally from tasks in CV, NLP, or multi‐modality generation. Those are tasks that human intelligence can do, although maybe imperfectly. For AI Biology, we desire it to help understanding what we cannot understand. Besides this fundamental difference, biological systems are high‐dimensional, noisy, context‐dependent, spatiotemporally dynamic, and constrained by evolutionary history and physical laws that we haven’t fully revealed. Available data reflect only a small fraction of the biological complexity. Consequently, there is a growing recognition that future AI Biology models must move beyond only adopting existing AI architectures. They must combine data‐based learning with knowledge‐driven design, with new AI models and principles. They should not merely fit existing data but should actively propose new experiments for extra data generation, and should improve through experimental and real‐world feedback. In this sense, biology may become not only an application domain for AI but also a source of new predictive, representational, and generative frameworks, model architectures, and learning principles.

### 4.2 The enduring role of experiments in AI Biology

While some people may worry that experimental biologists could be marginalized during the wave of AI biology, we believe that experimental biology will remain as a central part of AI Biology, though its roles and paradigms will evolve. As computational models generate hypotheses at scale, the critical task for experimentalists will shift from testing candidates one by one to designing most informative experiments—those that best guide AI models to more reliable hypotheses such as those that maximally constrain the model space or falsify key predictions. Bench science will not only provide data as the starting point for AI models, but will also provide feedback to reinforce models. Future biological studies will be a closed‐loop system: Existing experiment data nourish model building, model predictions inform experimental design, automated or high‐throughput platforms generate more data, and new data feedback to refine next‐generation models. The loop will not only produce new understandings on the biological system, but will lead to new ways to engineer the system.

This loop requires “AI‐ready” or “AI‐friendly” data—datasets that are not merely large but appropriately structured, including essential negative samples, spatiotemporal dynamics, perturbation outcomes, and causal relationships. Such data will have different properties from those targeted for being analyzed by human scientists. Wet‐lab validation serves as the ultimate touchstone for dry‐lab discoveries, particularly for generative models that propose novel sequences, structures, or cellular states. It should be realized that such validations may not be available or feasible for some results due to technical limitations or ethical restrictions. Proxy experimental systems such as organoids and corresponding validation techniques are more critically needed in the age of AI biology. The availability, efficiency, and reliability of experimental validation will likely become a bottleneck for the progress, making technology innovation in experiments as crucial as algorithmic development [27, 28].

### 4.3 Objects of modeling: From elements to systems

The objects of AI modeling in biology span multiple scales, from nucleotides and amino acids to genes, cells, tissues, organisms, and ecosystems. Future targets should not be at single levels but should be the multi‐scale biological system itself. A genome sequence is static, but its function is dynamic—varying across cell types, developmental stages, cell contexts, and environmental conditions—and shaped by evolutionary history. Effective AI models of genomes must ideally capture at least three dimensions: static sequence information, dynamic biological activity, and evolutionary constraints. In protein science, accurate structure prediction depends heavily on modeling evolutionary processes through multiple‐sequence alignments. In regulatory genomics, the challenge is not merely whether a sequence is functional but how it controls the timing, location, and level of gene expression. In modeling cells of multi‐cellular organisms, cells' response to stimuli and perturbations must be studied in tissue microenvironment contexts and relations among omics elements. This multi‐scale, dynamic, and evolutionary nature of biological systems distinguishes AI Biology from conventional AI applications and demands architectures specifically adapted to biological structure rather than borrowed wholesale from other domains.

### 4.4 Prediction, compression, and mechanistic understanding

A persistent tension in the field concerns the relationship between predictive accuracy and mechanistic insight. Current AI models have been shown to be powerful at compressing high‐dimensional biological data for extracting associations with predictive powers. For example, sequence models can learn motifs and high‐order patterns, structure predictors can capture spatial constraints, and spatial omics tools can identify cell‐type distributions and tissue domains. However, such compression is not equivalent to mechanism. A model may predict chromatin accessibility or protein structure with high accuracy without revealing how the underlying molecular process operates. Inferring mechanism and causality requires additional evidences: perturbation experiments, cross‐species generalization, consistency with known biochemical analyses, and the generation of testable hypotheses. Some researchers argue that an ideal model should do both: compressing data efficiently and revealing principles that can be experimentally interrogated. Others note that while we pursue both prediction and mechanistic understanding, we should not be fettered if we cannot achieve both. Even landmark achievements in protein structure prediction may not contribute directly to understanding the folding process, but this does not prevent the prediction model’s great contribution to the advancement of science. We should be open to the possibility that prediction and mechanism models may advance on separate tracks in parallel while we seek both goals.

## 5 CHALLENGES AND RECOMMENDATIONS

### 5.1 Beyond data, compute, and models

It is widely acknowledged that the success of AI depends on three pillars: data, computing power, and model architecture or algorithm. This triad is necessary but not sufficient for AI Biology. AI Biology requires at least two additional pillars.

First, biological knowledge. Biological data are not generic: They are structured by evolution, physical and chemical constraints, regulatory logic, experimental context, and technical and ethical limitations. Without proper knowledge, models may learn spurious correlations that fail to generalize. While the volume of biological data accumulation may be comparable with that of data used for training LLMs, the coverage of data relative to the intrinsic complexity of biological systems is far less than language data that covers human civilization. The success of fully data‐driven large AI models in NLP and CV may not be replicated in biology without effective involvement of biological knowledge and human inference.

Second, solid benchmarking, experimental feedback, and closed‐loop validation. Biological models must eventually be tested in living systems. The community has realized that the need of critical assessment is more crucial than ever before [28, 29, 30]. The absence of bridges between computational prediction and experimental validation on many biological questions is a major restrictive factor for AI biology. Data quality, bias, noise, and missing key information present persistent obstacles; biological objectives are often ambiguous; and the intrinsic complexity of living systems defies simple formulation. In LLMs, reinforcement learning with human feedback is essential to release the potential of pretrained models to successful real‐world application. But the ability of human feedback on unknown biology is limited. We need experimental technology advancement to provide proper feedback. The recent trend of automatic experimental systems provides a promising solution toward narrowing the gap. Scientists are also working on better benchmarking strategies and systems to avoid premature evaluations that may mislead the field [29, 30, 31].

### 5.2 Recommendations for the next generation

The coming of the AI era has triggered questions on what the new generation of biologists should be like. From a broad range of disciplinary backgrounds, we come up with a common prescription for training next‐generation scientists: avoid narrow identity as either “biologist” or “computational/AI researcher,” and cultivate deep literacy in both domains.

Biology trainees are encouraged to acquire early and rigorous training in mathematics, statistics, programming, and ML, but without sacrificing biological intuition—the ability to recognize important questions, evaluate data reliability, and design meaningful experiments. The training may not be directly “useful” in everyday jobs as AI tools and agents are becoming powerful on technical tasks, but understanding the principles is essential for correct and effective use of AI tools. Computational trainees are urged to study molecular biology, genetics, and experimental logics seriously, and to resist treating biological data as mere instances of images, texts, or tables. In this sense, the AI era increases rather than reduces requirements for researchers. Educators and trainees need to work together to meet the requirements efficiently, probably through the creative use of AI tools.

Beyond knowledge and technical skills, an emphasis is on problem‐driven inquiry. The most valuable discoveries will likely come not from applying the largest model or the most‐cited model to a convenient dataset, but from researchers who can identify the right question, build a reliable bridge between computation and experiment, and produce knowledge or designs that survive validation in real biological systems. The capacity for such inquiry requires more critical thinking. AI‐assisted literature study may help to improve reading efficiency but may not help building up such ability if the tools are not utilized with critical thinking. More specifically, we highlight several capabilities as essential: ability to formulate problems clearly for interaction with AI systems, critical judgment to evaluate and filter model outputs, exploratory data analysis skills to identify patterns and outliers in collaboration with AI, and creativity in recognizing unexplored valuable regions of the scientific landscape.

## 6 CONCLUSION

AI Biology stands at a historic point. AI models have demonstrated predictive power at or near experimental accuracy for an expanding set of biological tasks. The greater challenge now is to transform predictive capacity into genuine understanding, reliable design, and desirable engineering. This will require not only algorithmic innovations but also the co‐evolution of data‐generating technologies, the integration of biological knowledge in model architectures, and the construction of closed‐loop systems that bind computation to experiment. The future of the discipline, no matter you call it “AI Biology” or “AI for Biology,” depends less on whether AI can surpass human intuition in isolated tasks, but more on whether the scientific community can cultivate a new generation of researchers who move fluidly between the computational and the experimental, treating neither as merely a service to the other.

## FUNDING INFORMATION

The National Natural Science Foundation of China (Grant Nos. 92470105, T2495270, 62225307, 32430017, and 32125009); National Key R&D Program of China (Grant Nos. 2025YFC3409303 and 2022YFC3400300).

## Data Availability

Data sharing is not applicable to this article as no datasets were generated or analyzed during the current study.
